# RCOVID19: Recurrence-based SARS-CoV-2 features using chaos game representation

**DOI:** 10.1016/j.dib.2020.106144

**Published:** 2020-08-07

**Authors:** Mohammad Hossein Olyaee, Jamshid Pirgazi, Khosrow Khalifeh, Alireza Khanteymoori

**Affiliations:** a Faculty of Engineering, Department of Computer Engineering, University of Gonabad, Gonabad, Iran; b Department of Electrical and Computer Engineering, University of Science and Technology of Mazandaran, Behshahr, Iran; c Department of Biology, Faculty of Sciences, University of Zanjan, Zanjan, Iran; d Department of Computer Engineering, University of Zanjan, Zanjan, Iran

**Keywords:** SARS-CoV-2, Nonlinear analysis, Coordinate series, Chaos game representation, Recurrence quantification analysis

## Abstract

Severe acute respiratory syndrome coronavirus 2 (SARS-CoV-2) is responsible for the COVID-19 pandemic. It was first detected in China and was rapidly spread to other countries. Several thousands of whole genome sequences of SARS-CoV-2 have been reported and it is important to compare them and identify distinctive evolutionary/mutant markers. Utilizing chaos game representation (CGR) as well as recurrence quantification analysis (RQA) as a powerful nonlinear analysis technique, we proposed an effective process to extract several valuable features from genomic sequences of SARS-CoV-2. The represented features enable us to compare genomic sequences with different lengths. The provided dataset involves totally 18 RQA-based features for 4496 instances of SARS-CoV-2.

**Specification Table**

**Table:** 

Subject	Genetics, genomics and molecular biology
Specific subject area	Bioinformatics, Sequence analysis, Nonlinear analysis
Type of data	Table, Excel file(18 columns, 4496 rows)
How data were acquired	NCBI - Gene bank- SARS-CoV-2 https://www.ncbi.nlm.nih.gov/genbank/sars-cov-2-seqs/
Data format	Raw and Analyzed.
Parameters for data collection	Using MATLAB 2015a as well as CRP Toolbox 5.22.
Description of data collection	The genomic sequences were downloaded in the fasta format from NCBI. The coordinate series as well as the obtained features were generated by using Matlab and CRP Toolbox, respectively.
Data source location	Machine learning and bioinformatics lab, University of Zanjan
Data accessibility	The entire dataset is published in the Mendeley repository. Direct URL to data: https://data.mendeley.com/datasets/m3s26wghdz/2 The source code of generating data can be found in this address: https://github.com/mholyaee/RCOVID-19

**Value of the data**

• The dataset can be used by those working in bioinformatics and Artificial intelligence. Because they can apply machine learning methods to assess genomic information.
• The proposed dataset can be used for clustering and classification of SARS-CoV-2 genomes.
• The dataset can be effectively used for the investigation of the genetic diversity of SARS-CoV-2 genomic sequences.
• The dataset involves features that enable us to compare genomic sequences with different lengths.

## 1 Data description

A new coronavirus named SARS-CoV-2 appeared from Wuhan in China and has spread rapidly to the other provinces of China as well as the other countries. According to the situation report of the World Health Organization (WHO), as of 4 June 2020, more than six million cases of COVID19 have been confirmed around the world [1]. On 5 January 2020, the whole genome sequence of SARS-CoV-2 was provided and so far, several thousands of whole genome sequences have been presented [2]. Investigating these available nucleotide sequences provides an insight into its evolutionary similarity with other viruses as well as novel mutations. Indeed, it can provide valuable knowledge about designing vaccines and providing drugs [3].

**Fig. 1:**
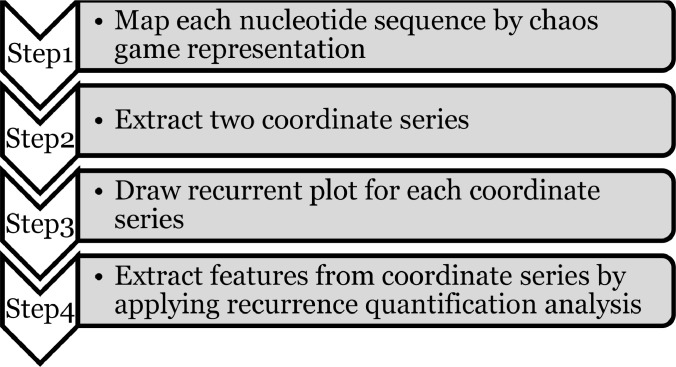
Diagram of extracting features.

For this aim, it is necessary to extract insightful features to describe the nucleotide sequences. In this work, according to the diagram represented in Fig. 1, several recurrence-quantification-based features are extracted from the nucleotide sequences. Recurrence quantification analysis (RQA) is a powerful nonlinear method which can propose representative features. This technique successfully aids us to compare biological sequences with different lengths [4,5].

In this paper, we introduce a new dataset which involves efficient nonlinear features related to genomic sequences of SARS-CoV-2. For this aim, the nucleotide sequences of 4496 variants of SARS-CoV-2 virus are gathered. The collected genomic sequences are publicly available in the National Center for Biotechnology Information (NCBI). As can be seen in Fig. 1, each nucleotide sequence is transformed into 2D space by applying chaos game representation. According to this map, all details of the input sequence are preserved. Next, the obtained picture is decomposed into two coordinate series which contain the position of points in the picture.

In the next step, recurrence plot (RP) as a powerful technique is used which illustrates recurrent properties in the coordinate series. In the final step, by applying recurrence quantification analysis (RQA), from each extracted coordinate series, 9 features are provided and totally 18 (2  ×  9) features will be extracted. The extracted features are described below:

(1) $$REC=\left(\text{Number}\;\text{of}\;\text{recurrence}\;\text{points}\right)/{N_{m}}^{2}$$

This measure describes the density of recurrence points in the RP. The second feature (DET) describes the amount of determinism which is gained as the ratio of the number of points constructing diagonal lines to all the recurrence points.

(2) DET=(Numberofpointsindiagonallines)/(Numberofrecurrentpoints)

*L_max_* and *L_mean_* are the next features which are the length of the longest diagonal line in RP and the mean of diagonal lines, respectively. ENT is the Shannon information entropy which describes the diversity of diagonal lines. This measure is computed as below:

(3) $$ENT=\;-\sum_{k=l_{min,p\left(k\right)\neq 0}}^{l_{max}}p\left(k\right)\log\left(p\left(k\right)\right)\;$$

In the above relation, *l_min_* is the minimum length of diagonal lines in the RP. Moreover, *p*(*k*) is obtained as below:

(4) p(k)=(Numberofdiagonallineswithlengthk)/(Numberofthediagonalline)

The next feature is laminarity which is computed as below:

(5) LAM=100×(Numberofpointsinverticallines)/(Numberofrecurrentpoints)

Trapping time (TT) is the next measure which equals the average length of vertical line structures. *V_max_* is the other feature which is the maximum length of the vertical lines in RP. Finally, the last feature is *C_v_* which is the average of the local clustering coefficient. This measure gives the probability that two neighbors of any state are also neighbors and is obtained as below:

(6) $$c_{v}=\sum_{i,j=1}^{N}\frac{RP_{v,i}RP_{i,j}RP_{j,v}}{k_{v}\left(k_{v}-1\right)}$$

In the above formula, *k_v_* is the degree centrality for node v which yields the number of its neighbors.

**Fig. 2:**
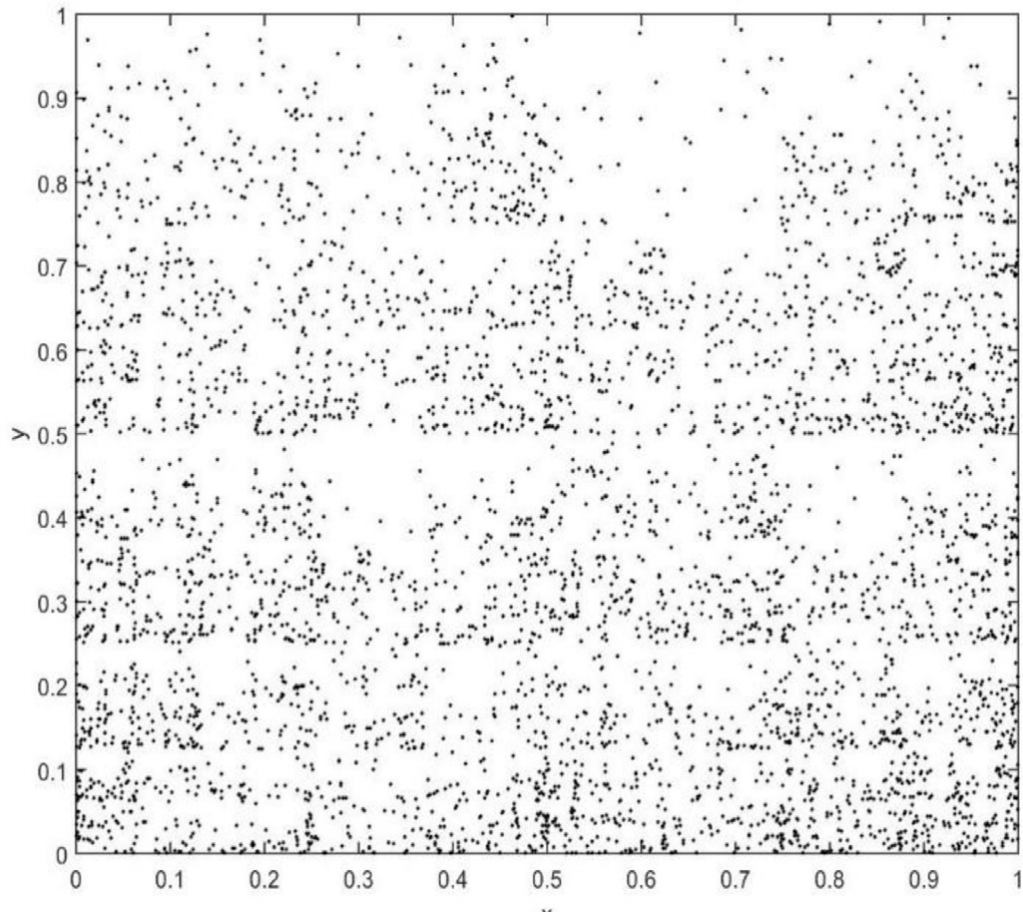
The CGR of viral genome MT503004.

**Fig. 3:**
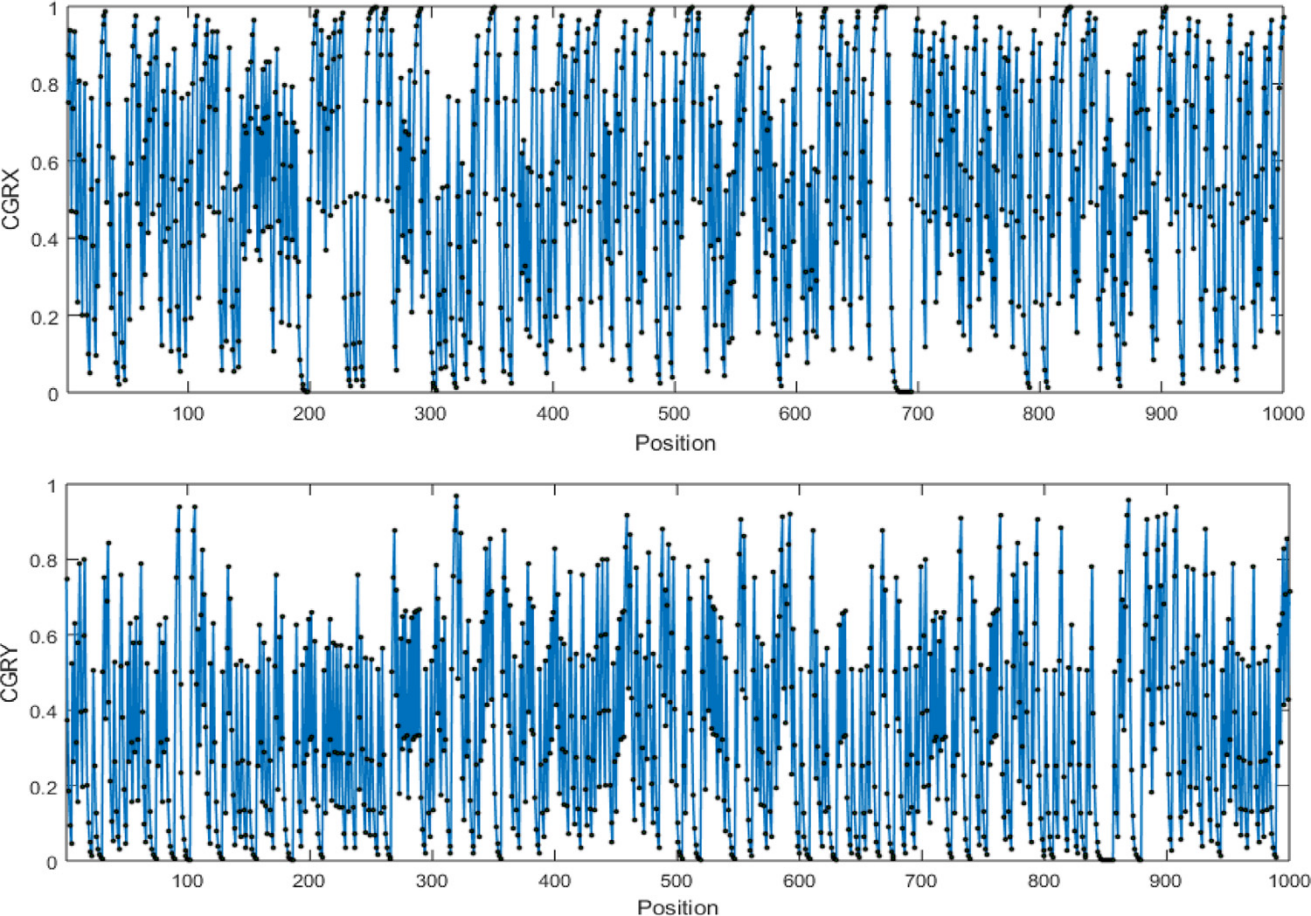
Two coordinate series extracted from CGR plot of Fig. 2.

**Fig. 4:**
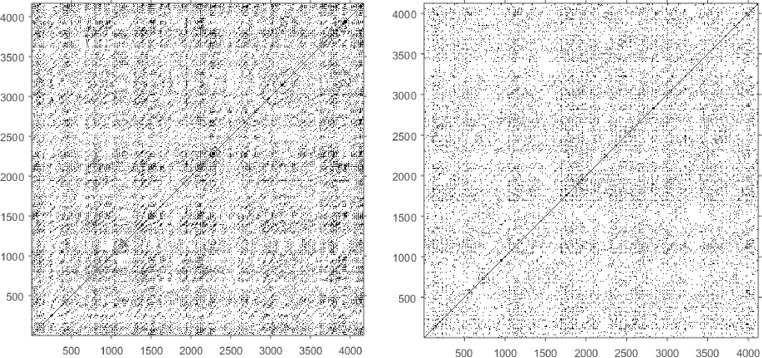
The corresponding recurrence plots for the coordinate series in Fig. 3. The Former is related to CGRX and the latter is the recurrence plot of CGRY.

The files of the dataset are represented in two folders named as “RQA” and “CGR”. The first involves two excel files which are named “GenomeInfo.xlsx” and “RQADataset.xlsx”. The former contains the information of nucleotide sequences which are GenBank accession, strain name, sequence length, and, nucleotide sequence. The latter is a table with 4496 rows and 18 columns which contains the extracted features for collected instances. The “CGR” folder contains raw information of viruses such that for each virus, a text file is stored which includes the point's positions of its CGR.

## 2 Experimental design, materials, and methods

As explained above, providing the dataset includes several steps. In this section, each part is reviewed with more details.

### 2.1 Chaos game representation

Chaos game representation (CGR) is an interesting map which transforms an input sequence into a two-dimensional space. The result of this map is a picture which reveals the hidden subsequence structures [6], [7], [8]. Let $S=(s_{1},s_{2},\ldots ,s_{N})$ be a given nucleotide sequence. Since it is composed of four kinds of letters (A,T,C, and G), the resulted map is a square [0, 1] × [0, 1] such that each vertex corresponds to a letter. The first point is located halfway between (0.5, 0.5) as the center of the square and the vertex equals the first letter. Each letter *s_i_* is iteratively mapped to a unique point halfway between the previous point and the vertex matching with *s_i_*. Fig. 2 demonstrates the resulting plot for MT503004.

It is interesting to note that like current implementations, it is supposed that the input sequence includes four alphabets and the other codes such as R and Y are omitted.

Since direct investigation of the obtained plot is a challenging task, according to the coordination of each point i.e. x and y, the CGR plot is decomposed into two coordinate series named CGRX and CGRY. Fig. 3 shows a part of the extracted coordinate series relating to the CGR plot of Fig. 2.

### 2.2 Recurrence plot

Recurrence is an essential feature of dynamical systems which emerges in the phase space [9]. Recurrence plot (RP) as a graphical tool enables us, for a given time series, to detect patterns of recurrence. In fact, RP is an *N_m_* × *N_m_* matrix; *N_m_* is the number of points in the phase space with dimension m and each entry of the matrix is 0/1. When one element (*RP*[*i, j*]) equals 1, it means the corresponding states of the two time points i and j are close. Fig. 4. Illustrates the RPs for the two coordinate series shown in Fig. 3.

## Declaration of Competing Interest

The authors declare that they have no known competing financial interests or personal relationships that could have appeared to influence the work reported in this paper.

## Appendix Supplementary materials

Image, application 1
